# The Role of Bronchoalveolar Lavage in the Diagnosis of COVID-19 Pneumonia: A Report of Three Cases

**DOI:** 10.7759/cureus.41145

**Published:** 2023-06-29

**Authors:** Fernando Poli De Frias, Rafael Cardenas Castillo, Maria F Perez Garcia, Ali Al-Himyary

**Affiliations:** 1 Internal Medicine, Mount Sinai Medical Center, Miami Beach, USA; 2 Pulmonary, Critical Care and Sleep Medicine, Baylor College of Medicine, Houston, USA; 3 Pulmonary, Critical Care and Sleep Medicine, CHI St. Luke’s Health, Baylor St. Luke’s Medical Center, Houston, USA

**Keywords:** bronchoalveolar lavage (bal), false-negative nasopharyngeal swab, acute respiratory syndrome, multifocal pneumonia, covid-19, sars-cov-2 rt-pcr

## Abstract

Severe acute respiratory syndrome coronavirus 2 (SARS-CoV-2) initially infects and replicates in epithelial cells of the nasopharynx where there are relatively high levels of angiotensin-converting enzyme 2 receptor, which correlates with the highest sensitivity time point of the nasopharyngeal swab (NPS) real-time polymerase chain reaction (RT-PCR) during the first week, with subsequent decline thereafter. As viral shedding progresses throughout the respiratory tract, the virus can be detectable for up to 30 days in bronchoalveolar fluids. This report presents three cases of acute respiratory distress in the setting of multifocal pneumonia, with multiple false-negative NPS SARS-CoV-2/RT-PCR but positive SARS-CoV-2/RT-PCR in bronchoalveolar lavage (BAL) samples. Molecular RT-PCR testing remains the gold standard in the diagnosis of SARS-CoV-2 infection. However, the diagnostic accuracy of NPS RT-PCR may be affected by several factors. SARS-CoV-2/RT-PCR in BAL samples increases the diagnostic yield for coronavirus disease 2019 pneumonia; however, it is not widely available in many institutions and can be clinically challenging to perform. A multimodal approach is required for prompt diagnosis, especially in patients with a progressive disease, where a delay in therapy can be clinically detrimental.

## Introduction

Severe acute respiratory syndrome coronavirus 2 (SARS-CoV-2) is an enveloped, positive-sense, single-stranded RNA virus capable of infecting humans and other mammals. It is the causative agent of coronavirus disease 2019 (COVID-19). The disease initially presented as a series of community-acquired viral pneumonia cases in the city of Wuhan, China, in December 2019 and has since spread worldwide as a rapidly evolving pandemic, causing millions of infections, deaths, and unprecedented global societal and economic disruptions. COVID-19 should be considered primarily in patients with fever and upper or lower respiratory tract symptoms, who resided in or have recently traveled to high-incidence regions, or who have had recent close contact with a confirmed or suspected case of COVID-19. It has a wide range of clinical presentations, ranging from asymptomatic or mild upper respiratory tract infections to mild and severe pneumonia, acute respiratory distress syndrome, and, ultimately, multiorgan failure and death [1]. Current National Institutes of Health guidelines for therapy in hospitalized patients include remdesivir in patients at high risk of developing severe COVID-19 pneumonia and dexamethasone when supplemental oxygen is required. Additionally, baricitinib or tocilizumab should be added despite being on first-line therapy with increasing oxygen requirements or severe inflammation [2]. As of today, COVID-19 infections have caused over six million deaths worldwide, with over 1,000 daily hospitalizations in the United States.

SARS-CoV-2 is an enveloped β-coronavirus with a genetic sequence similar to SARS-CoV-1 and MERS-CoV. However, some structural differences in its surface proteins, especially those related to the coronavirus spike (S) protein, confer greater affinity to SARS-CoV-2 to bind different host receptors and invade cells, especially the angiotensin-converting enzyme 2 receptor (ACE2-r), highly expressed in the nasopharynx epithelium [3]. SARS-CoV-2 real-time polymerase chain reaction (RT-PCR) from nasopharyngeal swab (NPS) specimens remains the gold standard for COVID-19. In addition, chest computed tomography (CT) scan has been widely used as a diagnostic and follow-up procedure since the beginning of the pandemic. The initial CT scan findings in patients include bilateral multi-lobar ground-glass opacification, most commonly in the lower lobes, which extends throughout the lungs as the disease stage progresses [4]. However, NPS SARS-CoV-2/RT-PCR has its limitations, and the false-negative rates can be as high as 30% [5,6].

In the following report, we describe the presentation and clinical course of three patients with acute respiratory distress in the setting of extensive COVID-19 multifocal pneumonia, with serial negative SARS-CoV-2 results on NPS, but positive on broncho-alveolar lavage (BAL) specimens.

## Case presentation

Case one

A 58-year-old Caucasian male with a medical history of hypertension, without smoking or recent travel history, presented to the emergency room (ER) with episodes of an objective fever at 101°F, body aches, cough, and shortness of breath. Two NPS SARS-CoV-2/RT-PCR tests were negative at this time. The patient was diagnosed with atypical pneumonia and was discharged home on symptomatic treatment and azithromycin. Due to worsening symptomatology, he returned to the ER one week later with shortness of breath, requiring 3 L/minute of supplemental oxygen by a nasal cannula (NC). Two additional NPS SARS-CoV-2/RT-PCR and influenza PCR tests were negative. He was hospitalized and started on broad-spectrum antibiotics.

A transfer to our facility was arranged five days later after the initial presentation for a higher level of care, considering episodes of fever and progressing hypoxia. Upon arrival, his body temperature was 98.6°F, blood pressure was 144/79 mmHg, heart rate was 84 beats/minute, and respiratory rate was 33 breaths/minute, with oxygen saturation of 91% on 15 L/minute high-flow nasal cannula (HFNC). Physical and neurological examinations were unremarkable except for bilateral lung crackles. Laboratory workup revealed leukocytosis, hypokalemia, elevated liver enzymes, and elevated inflammatory markers (Table 1).

**Table 1:** Summary of laboratory workup for all three patients at admission and before discharge. CBC: complete blood count; WBC: white blood cell; RBC: red blood cell; MCV: mean corpuscular volume; MCH: mean corpuscular hemoglobin; MCHC: mean corpuscular hemoglobin concentration; BUN: blood urea nitrogen; EGFR: estimated glomerular filtration rate; AST: aspartate aminotransferase; ALT: alanine aminotransferase; CRP: C-reactive protein; LDH: lactate dehydrogenase; INR: international normalized ratio; PT: prothrombin time; PTT: partial thromboplastin time; SARS-CoV-2: severe acute respiratory syndrome coronavirus 2; RT-PCR: real-time polymerase chain reaction; NPS: nasopharyngeal swab; BAL: bronchoalveolar lavage; ND: not done

Laboratory test (ranges)	Admission			Discharge		
	Case one	Case two	Case three	Case one	Case two	Case three
CBC						
WBC (4.5–11.0 × 10^3^/mm^3^)	21.9	4.1	4.1	6.2	10.7	6.5
RBC (3.9-5.8 × 10^6^/µL)	4.22	6.39	4.17	3.8	5.03	4.27
Hemoglobin (13–18 g/dL)	12.6	16.2	12.4	11.5	12.6	12.3
Hematocrit (37.0–49.0%)	36.6	49.9	37	35.4	39.8	37.3
MCV (78–100 µm^3^)	86.7	78.11	88.7	92.4	79.1	87.4
MCH (25.0–35.0 pg/cell)	29.9	25.4	29.7	30	25	28.8
MCHC (31–37 g/dL)	34	32.5	33.5	32.5	31.7	33
Platelets (130–400 × 10^3^/µL)	277	107	154	213	362	328
%Neutrophils (45–75%)	ND	61	80	65	ND	ND
%Lymphocytes (16–46%)		30	8	25		
%Monocytes (4–11%)		8	11	8		
%Eosinophils (0–5%)		0	0	2		
%Basophils (0–3%)		1	0	0		
%Immature granulocytes (<1%)		1	1	1		
Metabolic profile						
Sodium (135–145 mmol/L)	136	135	128	138	140	133
Potassium (3.4–5.0 mmol/L)	3.3	4	4.8	3.8	3.9	4.5
Chloride (95–108 mmol/L)	99	100	101	101	108	108
CO_2_ (20–32 mmol/L)	25	26	18	26	25	15
BUN (8–25 mg/dL)	10	10	30	13	16	35
Creatinine (0.8–1.1 g/day)	0.92	1.02	2.12	0.69	0.77	1.66
EGFR (>60 mL/minute/1.75m^2^)	84	80	31	118	111	42
Glucose (70–100 mg/dL)	141	98	106	106	100	110
Calcium (8.5–10.5 mg/dL)	8	8.5	8	9	8.3	8.8
Alkaline phosphatase (45–115 U/L)	50	142	ND	41	128	ND
Total bilirubin (0.0–1.0 mg/dL)	0.5	0.6		0.9	0.4	
Protein total (6.3–8.3 g/dL)	7.7	6.4		6.3	6	
Albumin (3.1–4.3 g/dL)	3.8	3.8		3.6	3.2	
AST (10–40 U/L)	70	98		37	105	
ALT (10–55 U/L)	48	74		56	160	
Procalcitonin (<0.1 ng/mL)	0.2	0.17		ND	0.05	
CRP (<0.5 mg/dl)	28.36	3.8			0.54	
Ferritin (12–300 mg/dl)	3225.9	3134.22			1650	
LDH (<270 U/L)	918	411			388	
Coagulation						
INR (<1.3)	1.21	1	ND	1.05	1	ND
PT (9–13 seconds)	15	10.3		13.3	13.2	
PTT (25-39 seconds)	33.1	26.1		30.6	ND	
D-dimer (<0.5 µg/mL)	2.37	0.63	0.57	2.3	0.38	
Fibrinogen (200–400 mg/dL)	748	ND	ND	458	618	
Microbiology						
Blood culture	Negative	Negative	(-) Coag., Staph species 2/2	ND	ND	Negative
Urine culture	Negative	Negative	Negative	ND	ND	ND
Sputum culture	Negative	Negative	Negative	ND	ND	ND
Fungus culture	Negative	Negative	Negative	ND	ND	ND
Bronchial culture	Negative	Negative	Negative	ND	ND	ND
SARS-CoV-2/RT-PCR NPS	Negative ×4	Negative ×4	Negative ×4	ND	ND	ND
SARS-CoV-2/RT-PCR BAL Sample	Positive	Positive	Positive	ND	ND	ND
Serology panel (Ab)						
Influenza A/B virus, M. pneumoniae, Aspergillus, Blastomyces, Coccidioides, Histoplasma	Negative	Negative	Negative	ND	ND	ND
Molecular (PCR)						
C. pneumoniae, human metapneumovirus, influenza A/B virus, parainfluenza virus 1/2/3, rhinovirus, adenovirus	Negative	Negative	Negative	ND	ND	ND

**Figure 1:**
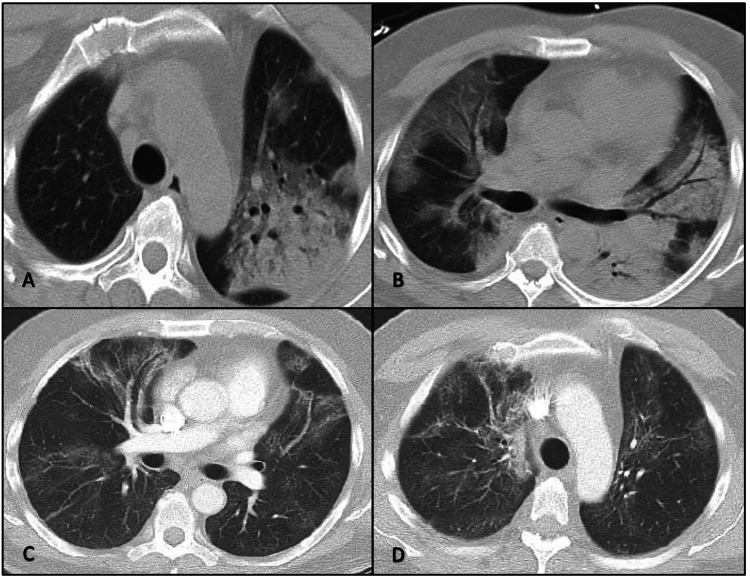
A and B: CT scan of the chest without contrast showing patchy and consolidative airspace opacities with air bronchograms and peripheral ground glass in the left upper lobe and bilateral lower lobes. C and D: CT scan of the chest with contrast showing improvement in opacities bilaterally after proper treatment with residual ground glass bilaterally.

Additionally, two NPS SARS-CoV-2/RT-PCR and a broad respiratory panel PCR were negative. A CT scan of the chest was concerning for multifocal pneumonia, for which he was started on empiric coverage with meropenem (1 g/TID) and vancomycin (1.5 g/QD) (Figure 1, Panels A, B).

On the next day after admission, due to tachypnea and increased oxygen requirements despite HFNC, a BAL was performed, and the SARS-CoV-2/RT-PCR was positive. After the procedure, the patient required mechanical ventilation due to a severe episode of cough with desaturation during the procedure. Subsequently, the patient was enrolled in a clinical trial receiving remdesivir plus tocilizumab/placebo (remdesivir for 10 days and one dose of tocilizumab/placebo). Additionally, he was started on dexamethasone (6 mg/QD) for 10 days. Ventilatory parameters progressively improved, and the patient was extubated five days later. A repeat CT scan of the chest with contrast revealed interval resolution of prior patchy opacities, and given his clinical improvement, the patient was discharged home (Figure 1, Panels C, D).

Case two

A 42-year-old Caucasian male healthcare worker with a medical history of hyperlipidemia initially exhibited cough, fever, and weakness for over three days. Two outpatient NPS SARS-CoV-2/RT-PCR tests were negative. Due to the persistent symptoms, he presented to the ER five days later, where a third NPS SARS-CoV-2/RT-PCR was negative. The patient was diagnosed with community-acquired pneumonia and was discharged home with amoxicillin and doxycycline, later changed to levofloxacin due to lack of improvement.

**Figure 2:**
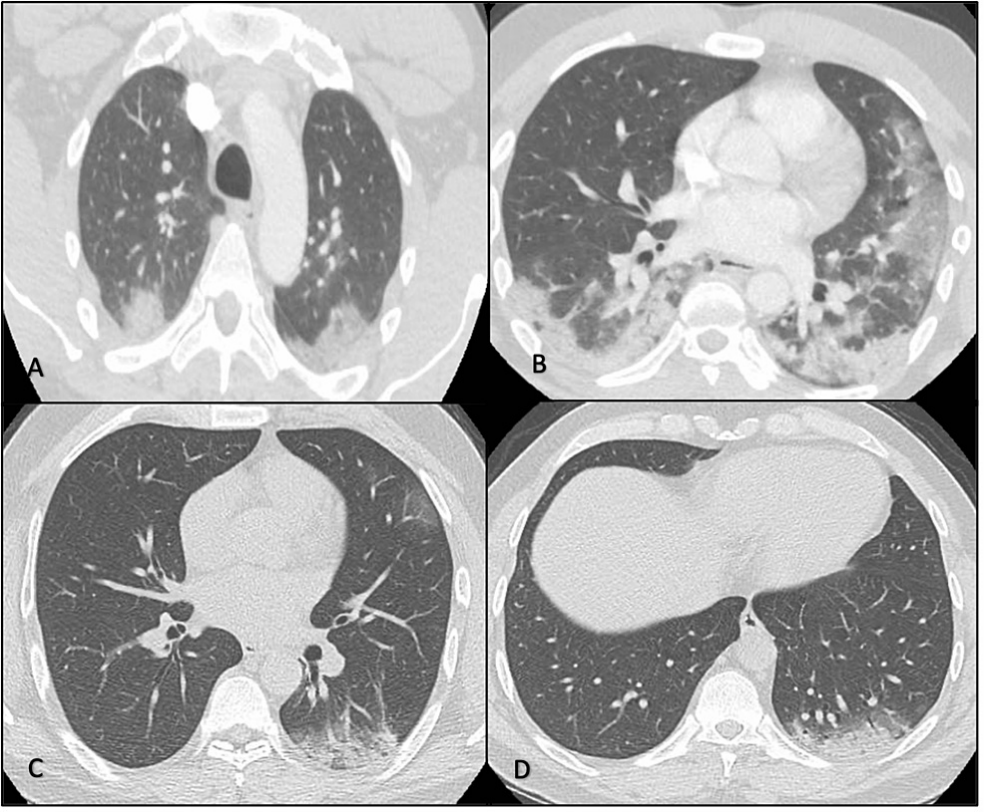
A and B: CT scan of the chest with contrast showing patchy and consolidative airspace opacities involving bilateral upper and lower lobes, and additional nonspecific ground glass in bilateral lower lobes. No pulmonary embolism was noticed. C and D: Interval resolution of prior findings with residual consolidation in the posterior left lower lobe.

The patient returned to the ER five days later with a worsening cough and shortness of breath. Physical examination showed a toxic appearance, tachycardia, and tachypnea with respiratory distress; oxygen saturation of 96% with 2 L/minute of supplemental oxygen requirement via NC; and decreased breath sounds with rales bilaterally, more prominent in the left upper middle fields of the lungs. A CT scan of the chest without contrast was concerning for multifocal pneumonia (Figure 2, Panels A, B).

He was admitted to the hospital with a presumptive diagnosis of atypical pneumonia and antibiotics coverage was changed to intravenous (IV) ceftriaxone (2 g/QD) and doxycycline (100 mg/BID) Initial blood work demonstrated leucopenia, thrombocytopenia, elevated liver enzymes, and elevated inflammatory markers (Table 1). NPS SARS-CoV-2/RT-PCR and a broad microbiologic respiratory PCR panel came back negative.

Subsequently, a diagnostic bronchoscopy and BAL revealed a positive SARS-CoV-2/RT-PCR test. The patient was started on dexamethasone (6 mg/QD) for 10 days. A CT scan of the chest revealed significant improvement (Figure 2, Panels C, D). The patient was discharged home given the resolution of his symptomatology.

Case three

**Figure 3:**
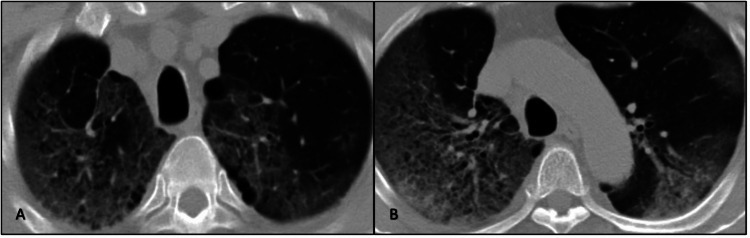
A and B: CT scan of the chest without contrast showing bilateral ground-glass opacities, more prominent and extensive on the right side, extending from upper to lower lobes. Additionally, patchy consolidations bilaterally with air bronchograms.

A 67-year-old Caucasian male, with a medical history of left renal pelvis urothelial carcinoma (status post-chemotherapy and left nephroureterectomy), hyperlipidemia, chronic kidney disease stage 3, and travel history one month before symptom onset, presented to the ER with complaints of intermittent fever of 101°F, body aches, chills, one episode of diarrhea, mild shortness of breath, and nonproductive cough in the last 10 days. An outside SARS-CoV-2/RT-PCR was negative. During the initial evaluation, body temperature was 99.1°F, respiratory rate was 24, with oxygen saturation of 92-95% on room air. Physical examination was only relevant for rales in the right hemithorax. Laboratory results revealed leucopenia and hyponatremia (Table 1). SARS-CoV-2/RT-PCR and broad microbiologic respiratory PCR panel were all negative. A CT scan of the chest showed extensive patchy ground-glass opacities in both lungs (Figures 3A, 3B).

Therefore, the patient was hospitalized with a presumptive diagnosis of community-acquired multifocal pneumonia. He was placed on 2 L/minute of oxygen flow through NC. IV azithromycin (500 mg/QD) and ceftriaxone (1 g/QD) were started.

On the second day after admission, blood cultures returned positive for coagulase-negative Staphylococcus species. Due to a lack of response to prior empiric treatment, antibiotic therapy was changed to IV vancomycin (1 g/QD) and meropenem (1 g/TID). Two days later, a repeat NPS SARS-CoV-2/RT-PCR was negative. The patient underwent a diagnostic bronchoscopy and BAL, with a positive SARS-CoV-2/RT-PCR. He was then started on dexamethasone (6 mg/QD).

**Figure 4:**
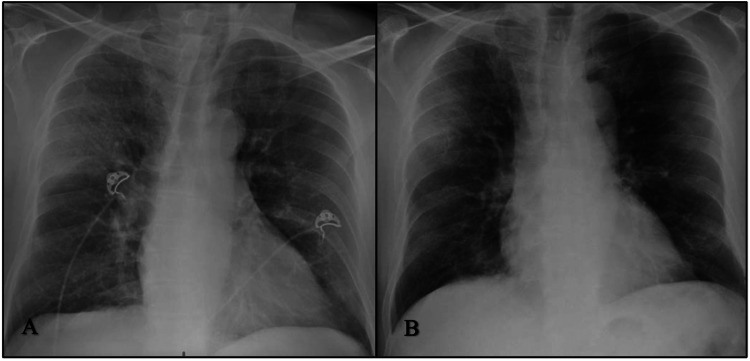
A: CXR from the admission day showing a right upper consolidation. B: CXR six days after admission showing improvement in interstitial thickening and airspace disease within the right lung. CXR: chest X-ray

The patient had substantial clinical and radiological improvement and was discharged home to continue dexamethasone for five more days (Figures 4A, 4B).

## Discussion

The SARS-CoV-2 virus initially infects and replicates in epithelial cells in the nasopharynx, where there are relatively high levels of ACE2-r, the targeted cell receptor in the host. This explains the initial higher viral load in upper respiratory tract specimens during the first week after symptom onset that correlates with the highest sensitivity of the NPS SARS-CoV-2/RT-PCR test, with an evident decline in the following weeks [7]. Large pooled analysis demonstrated that the false-negative rate was minimized to 20% four days after symptom onset, then began to increase again from 21% on day nine to 66% on day 21 [8]. As the viral shedding progresses throughout the respiratory tract, it infects bronchoalveolar epithelial cells and tissue-resident alveolar macrophages, generating an inflammatory response that becomes radiologically visible as bilateral pulmonary infiltrations [9,10]. At this time, NPS SARS-CoV-2/RT-PCR could become negative in the nasopharynx, although the virus could still be detected in BAL samples. This could suggest that temporal dynamics in viral shedding can differ from patient to patient and plays an important role in the diagnosis and disease spread. Based on inferred viral infectiousness profiles, it has been shown that viral shedding might begin five to six days before the appearance of the first symptom; however, specific viral-related factors such as viral mutations or host-related factors that could predispose to earlier viral shedding and nasopharyngeal clearance have not been identified [11].

The diagnostic yield of SARS-CoV-2/RT-PCR seems to be higher in the bronchoalveolar fluid compared to nasopharyngeal and sputum specimens, especially later in the course of illness [12,13]. Viral RNA decreases over time, and may become undetectable in NPS when the patient is tested later in the course of an illness; however, patients with severe disease had significantly higher viral loads and longer times of detection than patients with mild disease in sputum samples [14]. Interestingly, it has been proven that viral shedding in the lower respiratory tract can last almost 30 days in the median, which may be longer in severe diseases [15]. These findings make the BAL SARS-CoV-2/RT-PCR testing a great diagnostic tool in the context of a high index of suspicion in cases with multiple negative NPS tests. According to the World Health Organization guidelines, in patients with suspected COVID-19-associated pneumonia, negative specimens from the upper respiratory tract do not exclude the diagnosis, and additional lower respiratory tract samples are recommended [16]. However, BAL is not widely available, and internationally suggested indications should be followed, as this is a highly aerosolizing procedure, increasing airborne transmission and placing healthcare workers at particular risk of exposure and infection [17].

SARS-CoV-2/RT-PCR on NPS continues to be the gold standard for COVID-19 infections given its wide availability, time-efficient, cost-effectiveness, and easier technique. CT scans can also help to detect findings in the early infection of COVID-19 pneumonia, increasing diagnostic accuracy, especially in the context of typical clinical presentation despite false-negative SARS-CoV-2/RT-PCR on NPS [18,19]. The first week after symptom onset may be the optimal time for testing if the goal is to minimize false-negative results. Inadequate RT-PCR performance; sample deficiency or degradation; technical reasons related to collection, kit primers, probes, and fluorescence type; and the presence of RT-PCR inhibitors play a role in false-negative test results; however, repeated testing should minimize these factors. Hyperbilirubinemia, medical conditions with an excess of specific proteins (myoglobin, heme, monoclonal gammopathies, others), and specific drugs can affect PCR assay; however, these were not present in our cases [20]. Viral or host-related factors leading to early viral shedding and nasopharyngeal clearance can explain the repeated negativity in nasopharyngeal specimens. It is important to note that false-negative results can hinder the prevention and control of the epidemic, potentially contributing to a large proportion of community spread transmission, a big epidemiologic concern in a rapidly expanding pandemic. BAL samples increase the diagnostic yield of SARS-CoV-2/RT-PCR, as the viral load can persist longer in the lower respiratory tract compared to the nasopharynx.

## Conclusions

Our cases demonstrate that despite multiple negative NPS SARS-CoV-2/RT-PCR in different stages of the infection, the post-test probability remains high even with a negative test result. A high index of clinical suspicion, based on symptomatology, clinical course, and CT scan findings, should prompt consideration for obtaining more invasive studies such as diagnostic bronchoscopy with BAL whenever these can be timely and safely performed, especially in cases with progressive disease; however, it should not delay therapy, and the decision to treat empirically for COVID-19 pneumonia should be tailored to each specific case depending on the clinical course.

## References

[1] Wong CK, Wong JY, Tang EH, Au CH, Wai AK (2020). Clinical presentations, laboratory and radiological findings, and treatments for 11,028 COVID-19 patients: a systematic review and meta-analysis. Sci Rep.

[2] (2023). Coronavirus disease 2019 (COVID-19) treatment guidelines. https://www.covid19treatmentguidelines.nih.gov/.

[3] Antony P, Vijayan R (2021). Role of SARS-CoV-2 and ACE2 variations in COVID-19. Biomed J.

[4] Cevik M, Bamford CG, Ho A (2020). COVID-19 pandemic-a focused review for clinicians. Clin Microbiol Infect.

[5] Cevik M, Kuppalli K, Kindrachuk J, Peiris M (2020). Virology, transmission, and pathogenesis of SARS-CoV-2. BMJ.

[6] Woloshin S, Patel N, Kesselheim AS (2020). False negative tests for SARS-CoV-2 infection - challenges and implications. N Engl J Med.

[7] Wang W, Xu Y, Gao R, Lu R, Han K, Wu G, Tan W (2020). Detection of SARS-CoV-2 in different types of clinical specimens. JAMA.

[8] Kucirka LM, Lauer SA, Laeyendecker O, Boon D, Lessler J (2020). Variation in false-negative rate of reverse transcriptase polymerase chain reaction-based SARS-CoV-2 tests by time since exposure. Ann Intern Med.

[9] Pan Y, Zhang D, Yang P, Poon LL, Wang Q (2020). Viral load of SARS-CoV-2 in clinical samples. Lancet Infect Dis.

[10] Chu H, Chan JF, Wang Y (2020). Comparative replication and immune activation profiles of SARS-CoV-2 and SARS-CoV in human lungs: an ex vivo study with implications for the pathogenesis of COVID-19. Clin Infect Dis.

[11] He X, Lau EH, Wu P (2020). Temporal dynamics in viral shedding and transmissibility of COVID-19. Nat Med.

[12] Martinez RM (2020). Clinical samples for SARS-CoV-2 detection: review of the early literature. Clin Microbiol Newsl.

[13] Vannucci J, Ruberto F, Diso D (2020). Usefulness of bronchoalveolar lavage in suspect COVID-19 repeatedly negative swab test and interstitial lung disease. J Glob Antimicrob Resist.

[14] Zheng S, Fan J, Yu F (2020). Viral load dynamics and disease severity in patients infected with SARS-CoV-2 in Zhejiang province, China, January-March 2020: retrospective cohort study. BMJ.

[15] Buetti N, Wicky PH, Le Hingrat Q (2020). SARS-CoV-2 detection in the lower respiratory tract of invasively ventilated ARDS patients. Crit Care.

[16] (2023). World Health Organization (WHO). Coronavirus disease (COVID-19) technical guidance: early investigation protocol. https://www.who.int/emergencies/diseases/novel-coronavirus-2019/technical-guidance/early-investigation.

[17] Wahidi MM, Shojaee S, Lamb CR (2020). The use of bronchoscopy during the coronavirus disease 2019 pandemic: CHEST/AABIP guideline and expert panel report. Chest.

[18] Salehi S, Abedi A, Balakrishnan S, Gholamrezanezhad A (2020). Coronavirus disease 2019 (COVID-19): a systematic review of imaging findings in 919 patients. AJR Am J Roentgenol.

[19] Xie X, Zhong Z, Zhao W, Zheng C, Wang F, Liu J (2020). Chest CT for typical coronavirus disease 2019 (COVID-19) pneumonia: relationship to negative RT-PCR testing. Radiology.

[20] Mouliou DS, Gourgoulianis KI (2021). False-positive and false-negative COVID-19 cases: respiratory prevention and management strategies, vaccination, and further perspectives. Expert Rev Respir Med.

